# Diabetes and Abdominal Aortic Aneurysm: Is the Protective Effect on AAA Due to Antidiabetic Medications Alone, Due to the Disease Alone, or Both?

**DOI:** 10.26502/aimr.0169

**Published:** 2024-05-09

**Authors:** Gaithrri Shanmuganathan, Devendra K. Agrawal

**Affiliations:** 1 Department of Translational Research College of Osteopathic Medicine of the Pacific Western University of Health Sciences Pomona, California 91766

**Keywords:** AAA repair, Abdominal aortic aneurysm, Anti-diabetic drugs, Diabetes mellitus, Fluoroquinolones

## Abstract

Diabetes is a metabolic disease that may result in multiple microvascular and macrovascular diseases. Interestingly, many studies have demonstrated the inverse relationship between diabetes and the development and expansion of abdominal aortic aneurysm (AAA). One hypothesis is that the aortic wall stiffness resulting from hyperglycemia and advanced glycation end products could delay the development and growth of AAA. Other studies have proposed that the concurrent use of antidiabetic medications which promote anti-inflammatory cytokines while hindering pro-inflammatory cytokines may potentially be the reason for this protective effect of diabetes on AAA. Contrastingly, the presence of diabetes has been found to have a negative effect on the outcome of AAA following its repair which may be due to elevated blood glucose negatively affecting the healing process. The current literature has also demonstrated the negative impact of the use of fluoroquinolones on AAA. This comprehensive review critically reviewed and summarized the role of diabetes, anti-diabetes medications and fluoroquinolones on AAA, and on the effect of diabetes and certain anti-diabetes medications on outcomes following its repair.

## Introduction

Diabetes is a metabolic disease that occurs when the body is unable to make enough insulin, or when insulin produced by the body is not functioning the way it should to control the blood glucose level to baseline level, consequently causing hyperglycemia [1]. Hyperglycemia itself is defined as a fasting blood glucose level of >120mg/dL or 2-hour postprandial blood glucose level of >180mg/dL [2]. Generally, diabetes is categorized into four different types with varying underlying pathogenesis – diabetes mellitus type 1 (T1DM), diabetes mellitus type 2 (T2DM), gestational diabetes (GDM) and secondary diabetes [3]. While T1DM is typically characterized by the autoimmune degradation of pancreatic β cells involved in secreting insulin, T2DM tends to be caused by insulin resistance and deficiency because of poor diet and obesity, among other risk factors [3,4,5,6]. GDM on the other hand is characterized by hyperglycemia due to insulin resistance that is first diagnosed at 2nd or 3rd trimester of pregnancy, typically related to risk factors such as advanced age, gestational hypertension, and family history of diabetes [3,7,8]. If left untreated, hyperglycemia can lead to various short-term complications such as hyperglycemic crisis and life-threatening hypoglycemia, in addition to several long-term complications which include macrovascular diseases such as coronary artery diseases, cerebrovascular diseases, peripheral arterial diseases, and microvascular diseases such as diabetic retinopathy, diabetic neuropathy, diabetic foot, and diabetic nephropathy [3,4,9,10,11,12]. The ultimate concern with these long-term complications is that it can lead to increased mortality and morbidity [13,14,15]. While diabetes seems to have an adverse effect on most macrovascular diseases, there is one condition that has been found to have an inverse relationship with diabetes – abdominal aortic aneurysm (AAA) [16,17].

AAA is defined as the irreversible pathological dilation of the wall of the abdominal aorta of 3.0 cm or more [18, 19, 20]. Some of the many risk factors associated with AAA include populations in the Western Pacific region, males, smokers, elderly, and those with hypertension, obesity, hypercholesterolemia, and a family history of AAA [21, 22, 23]. However, recent studies have found that the rate of size-dependent aneurysm growth and rupture tends to be higher in female patients, therefore making it more fatal for them [24, 25]. The current proposed pathophysiology involved in the formation of AAA includes a combination of vascular inflammation, smooth muscle apoptosis, inflammatory processes involving cytokines, inflammatory cells, oxidative stress and the complement cascade, and extracellular matrix degradation [26, 27, 28, 29]. The current literature states that being diagnosed with diabetes tends to have a protective role against AAA [30]. One of the proposed reasoning behind the way in which diabetes protects from AAA formation is that the concurrent use of antidiabetic medications aids in modifying the pathophysiological process of AAA development which helps curtail its formation [31, 32, 33]. Another proposed idea is that diabetes could potentially change the macrophage metabolism, therefore protecting against AAA development [34, 35]. Additionally, it has also been suggested that the presence of cell division autoantigen 1 (CDA 1) protects against severe aneurysm [36]. The aim of this review was to critically review the current findings on the effect of diabetes and anti-diabetic agents on AAA formation, and the effect of diabetes on AAA following its repair. Additionally, this article also highlights the antibiotics that are contraindicated in patients with AAA.

## Method

A series of steps were taken to select published research findings in English language for this article on the effect of diabetes and anti-diabetic medications on the development and expansion of AAA. A comprehensive search was performed using database including Google Scholar, Pubmed, and NCBI. Search results included terms such as: “Diabetes”, “Protective effects of diabetes”, “Protective effects of anti-diabetes medications”, “Abdominal aortic aneurysm”, “Sulfonylureas”, “GLP-1 agonists”, “Metformin”, “DPP4-inhibitors”, “SGLT-2 inhibitors”, “Role of diabetes on AAA”, “Fluoroquinolones”. Duplicate articles, only abstracts, and non-English articles were excluded during the literature search following PRISMA guidelines.

### Pathophysiology of AAA

**Figure 1:**
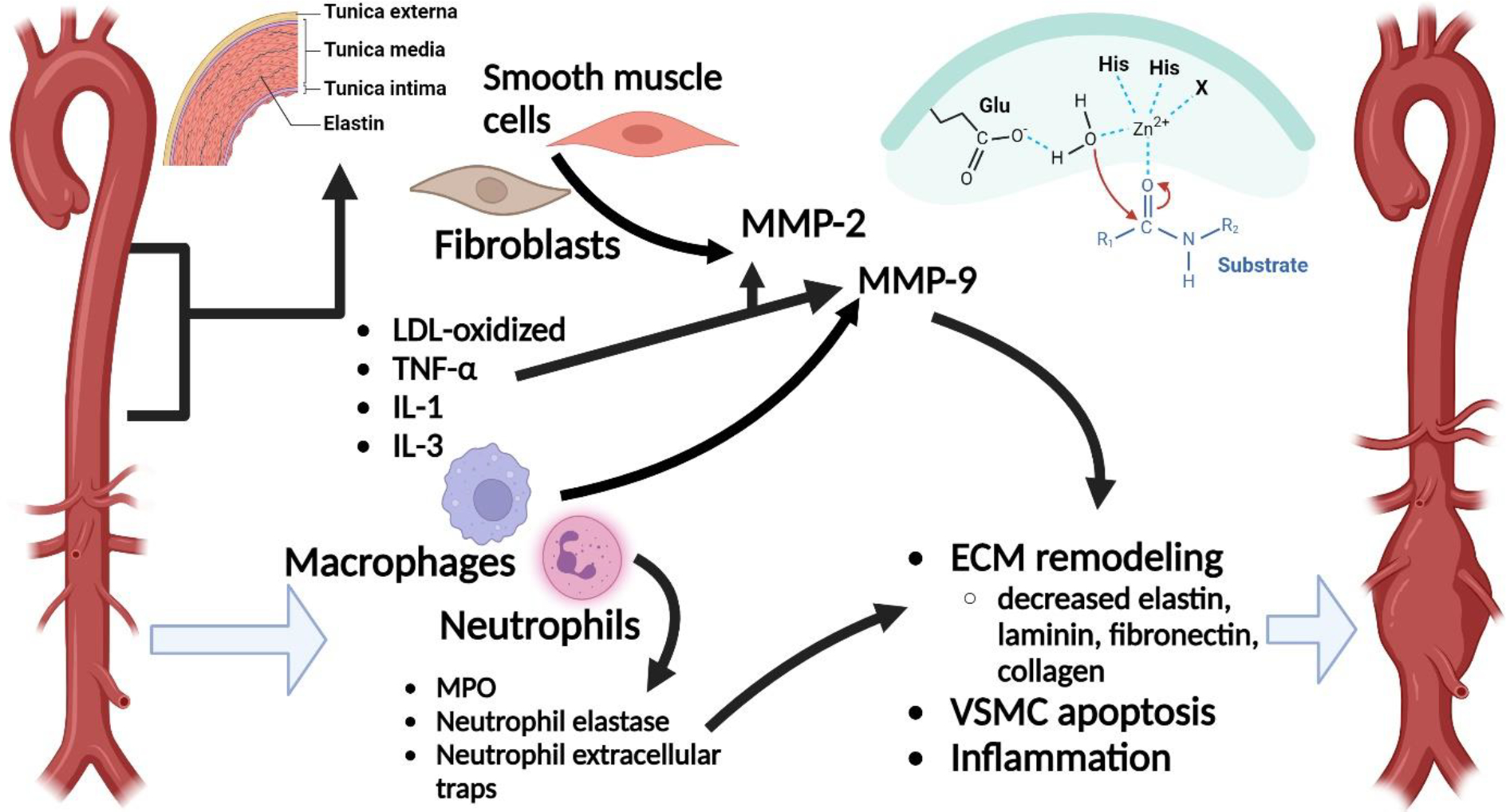
The multifactorial pathophysiology of AAA. MMP-2 is produced by smooth muscle cells and fibroblasts, and MMP-9 is produced by neutrophils and macrophages. MMPs are also produced by oxidized LDL, TNF-α, IL-1 and IL-3. MMPs, MPO, neutrophil elastase and neutrophil extracellular traps collectively lead to ECM remodeling, VSMC apoptosis and inflammation. These processes consequently lead to the formation of AAA.

The pathophysiology of abdominal aortic aneurysm is multifactorial (Figure 1). Currently, the understanding is that both inflammation and proteolytic degeneration of connective tissue proteins play a role in forming abdominal aortic aneurysm. Regarding to inflammation, both innate and adaptive immune cells play a major role [37]. Among all innate cells, the role of neutrophils in inflammation is multifaceted. It expresses azurophilic granules which contain myeloperoxidase, a type of peroxidase enzyme involved in oxidative stress [38]. In fact, studies have shown that a defect of myeloperoxidase (MPO) is associated with decreased AAA formation [38]. Besides that, neutrophils also produce neutrophil elastase, metalloproteinases, and neutrophil extracellular traps, especially when activated by lipopolysaccharides and cholesterol crystals [38,39]. The consequence of the production of these proteins is that it leads to further inflammation and vascular smooth muscle cell apoptosis, all of which lead to AAA [18,39].

In terms of proteolytic degeneration, oxidized LDL, TNF-α, IL-1 and IL-3 result in increased proteolytic enzyme such as matrix metalloproteinases (MMPs) in the aortic wall, causing a subsequent decrease in extracellular matrix protein including elastin, laminin, fibronectin, and collagen [40]. While there are many types of MMPs which play a role in the pathogenesis of AAA, two are found to be significantly upregulated [41,42,43]. This includes MMP-2 and MMP-9, formed by smooth muscle cells and fibroblasts, and by macrophages and neutrophils, respectively [40,42,43]. The consequence of these elevated MMP is a combination of extracellular matrix remodeling, vascular smooth muscle cell (VSMC) apoptosis, and inflammation [40,44].

As a result of inflammation and proteolytic degradation, the abdominal aortic wall loses its structural integrity, causing widening of the vessel. With the addition of elevated blood pressure which then causes mechanical stress on the wall of the aorta, there is bound to be further dilation and rupture of the abdominal aorta [45,46].

### Protective Role of DM on AAA

Many studies over the years have found that patients with diabetes have decreased development and growth of AAA. One study reported that patients with diabetes had significantly slower AAA growth rate (P<0.0001) compared to those without diabetes [16]. Additionally, this study also concluded that the AAA maximal transverse diameter (MTD) median growth of patients with diabetes was reduced by 37% [16]. While the growth pattern of AAA in both diabetic and non-diabetic group was found to be mostly linear, 40% of those in diabetic group was found to have an indeterminate growth pattern compared to 19% in non-diabetic group, further proving that diabetes slowed down the growth of AAA [16]. Another study analyzed data collected from mostly Mexican American patients and found the AAA expansion rate per month to be 65% lower in patients with diabetes compared to those without diabetes [47]. Specifically, the growth rate was 0.07mm per month and 0.21mm per month in diabetic and non-diabetic patients, respectively [47].

There are several theories suggested by current research regarding the protective role of diabetes against AAA development and growth. One idea is that chronic hyperglycemia results in a thicker aortic wall by stabilizing the collagen network. Similarly, it has also been postulated that hyperglycemia forms advanced glycation end products (AGE) which eventually crosslinks, forming a stable aortic wall [48]. On the other hand, other studies hypothesized that chronic hyperglycemia resulting from diabetes may allow for an increase in macrophage metabolism, causing it to have an anti-inflammatory effect, therefore protecting the walls of abdominal aorta [34, 35]. While these studies demonstrate the direct protective effect of hyperglycemia on AAA, other studies question if the concurrent use of hypoglycemic agents may be the real reason for protection.

### Antidiabetic Medications and AAA

#### (i) Metformin

After lifestyle modification, the current first-line anti-hyperglycemic medication for patients with Type 2 Diabetes (T2DM) is metformin [49]. This hypoglycemic agent acts in several ways to treat T2DM including through the decrease in low density lipoprotein levels, activation of 5’ AMP-activated protein kinase (AMPK) signaling which decreases hepatic gluconeogenesis, increased peripheral insulin release of skeletal muscle cells via the increase in tyrosine kinase activity and increased glucose uptake through glucose transporter type 4 (GLUT-4) carrier protein [50,51,52,53].

As for its relationship with AAA, metformin seems to have a beneficial effect. One retrospective study investigated the relationship between metformin exposure and AAA-related death in three groups including diabetic veterans treated with metformin, diabetic veterans not treated with metformin, and non-diabetic veterans [54]. It was found that the highest AAA-related death occurred in diabetic patients who did not receive metformin [54]. Similarly, a Swedish cohort study concluded there was a decrease in growth rate of small AAA in groups treated with metformin [55]. Specifically, the mean growth rate of AAA in metformin-treated patients was 1.1mm while the non-metformin-treated group was 1.6mm in one unadjusted and two adjusted groups (P<0.001, P = 0.005, P = 0.024, respectively) [55]. Similarly, using a mouse model, one research study demonstrated that metformin decreased the expression of Angiotensin II-produced proteins LC3B and Beclin1 and phosphorylation of PI3K/Akt/mTOR and NF-κB signaling pathways which are expressed in abdominal aortic tissues which typically played a role in autophagy and in the formation of AAA [56]. Another mouse model demonstrated the ability of metformin to block the macrophage-activated transformation of VSMCs from a contractive form to a synthetic form, reducing the progression of AAA [57]. Taken together, these findings support the notion that metformin plays a protective role in AAA.

#### (ii) SGLT2 inhibitor

Sodium–glucose cotransporter 2 (SGLT2) inhibitors such as canagliflozin, dapagliflozin, empagliflozin, and ertugliflozin are another class of hypoglycemic drug proven to be efficacious in treating diabetes [58]. The way in which this class of drugs treats diabetes is that it blocks SGLT2 proteins embedded in proximal convoluted tubules of kidneys, preventing the reabsorption of glucose into the body, which essentially keeps the blood glucose level well-regulated [58].

Current studies have demonstrated SGLT2 inhibitors to have a mitigative effect on AAA. One study proposed that this effect seems to be dose dependent. A higher empagliflozin dose of 3 mg/kg compared to lower dose at 1 mg/kg has been found to prevent formation and progression of AAA by decreasing Ang-II-induced degradation of elastic laminae, medial smooth muscle α-actin cells, thoracic and ascending aortic diameter expansion, macrophage infiltration and CD31+ capillary microvessel formation [59]. Additionally, mouse models treated with empagliflozin prior to Ang-II induction demonstrated decreased nuclear factor-κB (NF-κB) and p38 mitogen-activated protein kinase (p38 MAPK) in suprarenal aorta, both of which play a crucial role in the early phase of AAA development (P<0.05) [59]. Empagliflozin was also found to decrease levels of proinflammatory chemokines CCL-2 and CCL-5, metalloproteases MMP-2 and MMP-9, and VEGF, all of which are found to contribute to formation of AAA in humans [59]. Another study similarly demonstrated the decrease in progression of AAA after treatment of mouse model with 5 mg/kg dapagliflozin through the reduction in aortic inflammation and elastin degradation, preservation of medial smooth muscle cells, inhibition of macrophage infiltration through reduction of CD4+ T cells and B cells, and improvement in smooth muscle cell cellularity [60]. Like empagliflozin, dapagliflozin was also found to reduce aneurysmal angiogenesis, MMP-2 and MMP-9 levels, all of which play a significant role in AAA formation [60]. This newer study also demonstrated decreased medial smooth muscle cell loss when treated with 5 mg/kg dapagliflozin. Taken together, these findings support the beneficial role of SGLT2-inhibitors in preventing the development and progression of AAA.

#### (iii) Thiazolidinediones

Thiazolidinediones such as pioglitazone and rosiglitazone have been found to activate peroxisome proliferator-activated receptors (PPARs) present on various cells in our body including adipose cells, myocardial cells, and skeletal muscle cells, which results in increased insulin sensitivity and decreased triglycerides [61, 62, 63]. While their use in the treatment of T2DM seems to be as efficacious as other hypoglycemic agents, their use has declined due to studies showing an increased risk of heart failure after the use of this agent.

Regarding their role in AAA, there seems to be limited studies demonstrating the favorable effect of thiazolidinediones on AAA. However, one recent study did prove pioglitazone favorable in significantly reducing levels of MMP-9 by 50% in ex-vivo model of human mesenchymal stem cells isolated from AAA specimen, which is repeatedly shown to play a role in the pathogenesis of AAA [64].

#### (iv) DPP4 inhibitor

Currently, dipeptidyl peptidase IV (DPP IV) inhibitors including sitagliptin and saxagliptin are commonly used as a second-line treatment of T2DM, in conjunction with metformin or other hypoglycemic agents, especially if the use of metformin on its own has failed to treat hyperglycemia [65]. DPP IV inhibitors work by blocking the degradation of glucagon-like-peptide-1 (GLP-1) by DPP IV, which consequently decreases glucagon secretion while increasing insulin secretion [65]. Many studies have demonstrated the beneficial effect of DPP IV inhibitors on preventing the progression of AAA. Using a murine model, one study showed that DPP IV inhibitor can decrease extracellular signal-regulated kinase (ERK) and Akt function, mRNA expression of monocyte chemoattractant protein-1 (MCP-1) in VSMCs, aortic dilatation, elastin degradation, and macrophage infiltration, all of which play a role in AAA formation [66]. Another study compared patients with AAA and controls, demonstrating that DPP4 was found to be strongly expressed in media and adventitia, including in inflammatory infiltrates and neovessels found throughout AAA [67]. Furthermore, DPP4 was found to correlate with macrophage marker CD68 and T-cell marker CD4 in both media and adventitia [67]. Taken together, these findings support the idea that DPP4 inhibitors can be used as a potential pharmaceutical agent in AAA.

#### (v) GLP-1 agonist

GLP-1 is an incretin hormone produced by the pancreas, which typically binds GLP-1 receptors, causing increased insulin release which subsequently decreases glucose levels. GLP-1 agonists such as liraglutide and semaglutide, were engineered to mimic the function of GLP-1 hormone [68].

Using mouse models, studies have shown that GLP-1 agonists do have a protective effect against AAA formation [69]. Most recent studies demonstrated that this protective effect against AAA formation is more pronounced when treated early on [70]. This study found that mice which were treated with liraglutide early on had significantly greater reduction in ROS-produced malondialdehyde (MDA) in the wall of abdominal aorta than those treated later [70]. Additionally, there was significantly more production of anti-inflammatory cytokines Ym-1 and TGF-β in groups of mice treated with liraglutide early on compared to those treated later [70]. There was also significantly more production of proinflammatory cytokines TNF-α in earlier liraglutide-treated groups compared to later liraglutide-treated groups [70]. This study also demonstrated greater reduction in alpha-smooth muscle actin (α-SMA) protein expression in earlier liraglutide-treated groups [70]. The significance of this protein is that it is responsible for the proliferative effect of VSMC which subsequently promotes the formation of AAA. Taken together, these findings suggest that GLP-1 agonist itself protects against AAA but has a more prominent effect when utilized early on.

#### (vi) Sulfonylureas

Used as a first-line hypoglycemic agent in those who are unable to tolerate metformin, sulfonylureas such as glimepiride, tolbutamide, and glyburide work to decrease blood glucose levels by inhibiting the ATP-sensitive potassium channel, subsequently increasing endogenous insulin release [71, 72]. Sulfonylureas have also been found to play a protective role in AAA formation. This was demonstrated by a retrospective study using data of diabetes patients with AAA with or without rupture, obtained from the VA Health System between years 2003 and 2013 [73]. This study found that compared to the remaining cohort with an AAA expansion rate of 1.4mm/year, patients treated with sulfonylureas had a significantly lower expansion rate of 1.3mm/year (P <0.006) [73].

### Effect of DM and Anti-diabetic Agents on Outcomes following AAA Repair

There seems to be a negative association between diabetes and post-operative healing [74]. Post-operative hyperglycemic patients have generally been found to have more infection and greater mortality [74]. The greater risk of infection under hyperglycemic conditions can be explained by the impaired neutrophil function including production of reactive oxidative stress and decreased adherence, migration, phagocytosis, and bactericidal effects which subsequently negatively affects the healing process [74]. While diabetes has shown to have a protective effect on AAA, it does not seem to have the same beneficial effect on AAA following surgical repair [75]. A 10-year multicenter retrospective study conducted in France found that the prevalence of T1DM was associated with greater post-operative mortality in AAA patients after the first repair [75]. Interestingly, this association was not statistically significant when compared to T2DM [75]. Another retrospective study demonstrated that there was a difference in the effect of postoperative hyperglycemia on the patients’ post-operative progress and this depended on the type of repair done [76]. Specifically, it was found that patients who underwent endovascular repair had increased rates of infection and in-hospital mortality while those who underwent open AAA repair had increased chances of death [76]. Another study found similar results whereby of the 21,943 patients who met inclusion criteria, insulin-dependent diabetic patients undergoing open aortic aneurysm repair or endovascular aneurysm repair had higher rates of 30-day readmission, post-operative myocardial infarct, and at increased risk of mortality at 1-year, 3-year and 5-year follow-up [77]. Another study concluded that patients with diabetes who underwent endovascular repair had higher cardiovascular events and all-cause mortality compared to those without diabetes [78]. This was found to be due to diabetes in patients with an association with increased BMIs, tobacco use, triglyceride levels, lower extremity artery diseases and coronary artery diseases, all of which potentially increase cardiovascular events and mortality [78].

The current literature seems to be limited on findings concerning the effect of the various anti-diabetic medications on post-operative outcomes. However, one study did investigate the effect of metformin on mortality and complications following abdominal aortic aneurysm repair [79]. This retrospective study of 306 patients found a significant decrease in mortality (P = 0.019) and complications (P = 0.032) in post-AAA repair patients with diabetes treated with metformin. When compared with other groups including post-AAA repair patients with diabetes treated with other anti-diabetic medications and non-diabetic patients, this association seems to become nonsignificant [79]. Given that diabetes itself may not be completely cured after the surgical repair of AAA, it would be helpful to determine the effects that these other anti-diabetic medications may have post-repair.

### Drugs that are contraindicated in AAA: Fluoroquinolones

**Figure 2:**
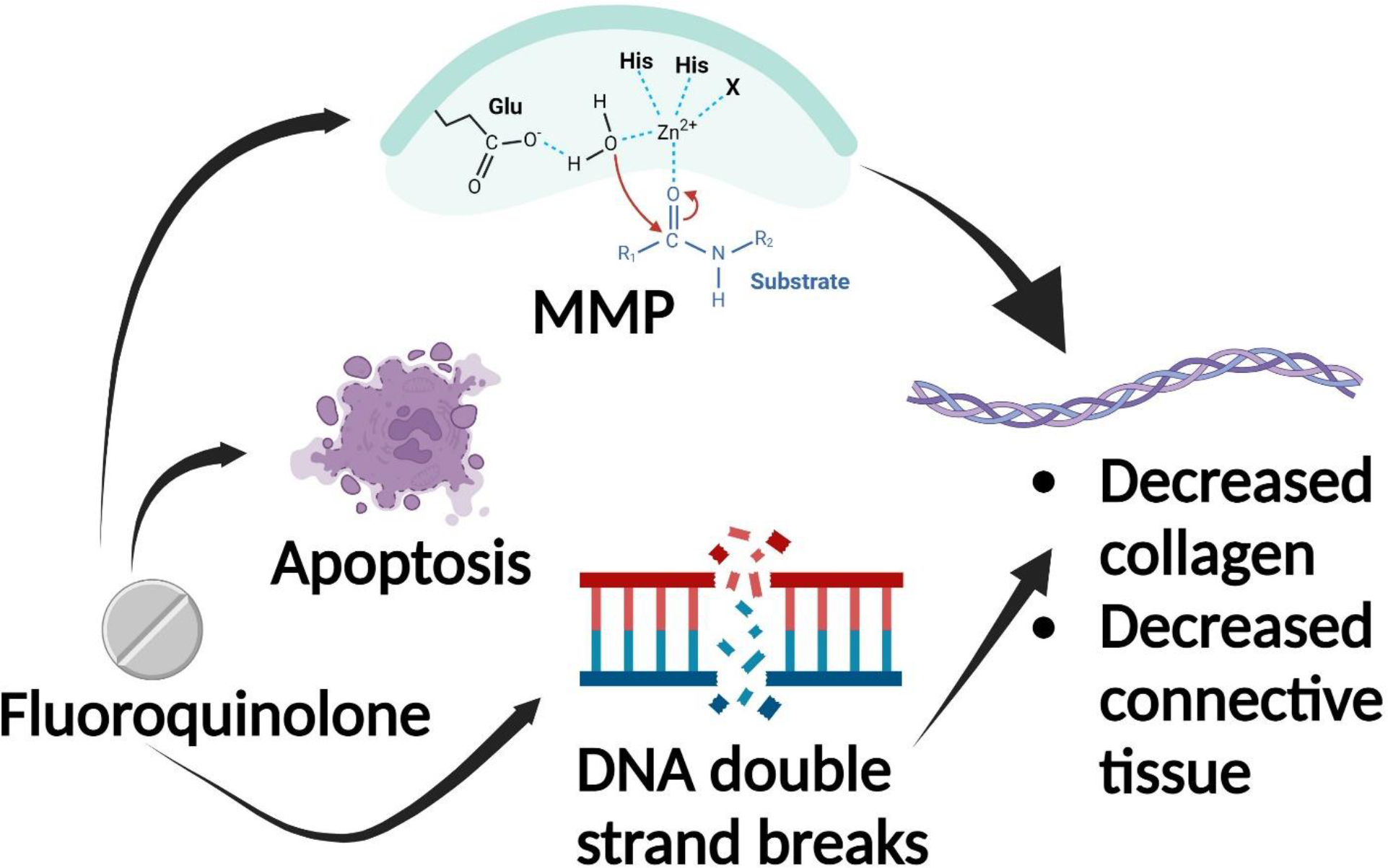
The use of Fluoroquinolone results in increased MMP, cellular apoptosis, and DNA double break strands which cause degeneration of collagen and connective tissue in the aortic wall. This results in AAA progression.

Many studies have found a relationship between the use of fluoroquinolones and formation of abdominal aortic aneurysm. In fact, some studies have found that the duration of fluoroquinolone use correlates with the development and severity of aneurysm (Figure 2). A study was conducted on sham-operated and aneurysm-induced animals to observe the effect of duration of ciprofloxacin use, a second-generation fluoroquinolone, on these groups. It was found that the diameter of aneurysm was greater in the group of animals which received ciprofloxacin for 4 weeks compared to animals which received saline only or ciprofloxacin for 2 weeks only [80]. Another meta-analysis found that the risk of developing aortic aneurysm was greater when the use of fluoroquinolones exceeded 14 days, with 60 days carrying the greatest risk [81]. This effect was more pronounced in older patients [81]. Another cohort study found a positive association between fluoroquinolone use and development of aortic aneurysm in patients of ages 35 years and over in the US, regardless of their risk factors, when compared to use of other antibiotics [82]. Additionally, studies have found that the 90-day incidence of AAA formation increases greatly with the oral method of administration [83].

The mechanism by which fluoroquinolone causes abdominal aortic aneurysm is thought to be through the degradation of collagen and connective tissue of the aortic wall because of increased MMP, decreased lysyl oxidase levels and increased apoptosis [84]. Specifically, a study done on mice models proposed that in addition to increasing the expression of MMPs, this antimicrobial plays a role in decreasing lysyl oxidase levels which is an important contributor to the assembly, stabilization and formation of elastin, collagen, and extracellular matrix [84]. Additionally, there was more DNA double strand breaks in the nucleus, along with the release of mitochondrial and nuclear into the cytosol of the aortic smooth muscle cells of mice which were treated with fluoroquinolone, indicating that apoptosis was greater in this group [84].

## Conclusion

Currently, the overall findings suggest a protective effect of diabetes on AAA but does not hold the same favorable effect after AAA repair. The literature also speculates that several anti-diabetic medications currently used in the management of diabetes also play a protective role against AAA. However, careful and case-controlled studies are warranted to distinguish whether diabetes in itself protects against AAA or does the concurrent use of anti-diabetes medications curtails the formation and growth of AAA. Additionally, antibiotics belonging to the Fluoroquinolone class have been found to worsen AAA through its mechanism on the collagen. The importance of delineating the role of diabetes and anti-diabetic medications in AAA would help providers effectively utilize current treatment options to slow down the time it takes for their patients to undergo AAA surgical repair while improving the prognosis of patients diagnosed with AAA.
